# Functional soft robotic composites based on organic photovoltaic and dielectric elastomer actuator

**DOI:** 10.1038/s41598-024-60899-6

**Published:** 2024-04-30

**Authors:** Ahmed Miguel Román Abolhosen, Shinyoung Lee, Kenjiro Fukuda, Takao Someya, Leobardo Hernández González, Jun Shintake

**Affiliations:** 1 Department of Mechanical and Intelligent Systems Engineering, The University of Electro-Communications, 1-5-1 Chofugaoka, Chofu, Tokyo 182-8585 Japan; 2 RIKEN Center for Emergent Matter Science (CEMS), 2-1 Hirosawa, Wako, Saitama 351-0198 Japan; 3 Thin-Film Device Laboratory, RIKEN, 2-1 Hirosawa, Wako, Saitama 351-0198 Japan; 4 Department of Electrical and Electronic Engineering and Information Systems, The University of Tokyo, 7-3-1 Bunkyo-Ku, Tokyo, 113−8656 Japan; 5 Escuela Superior de Ingeniería Mecánica y Eléctrica, Unidad Culhuacán, Instituto Politécnico Nacional, Col. San Francisco Culhuacán, Av. Santa Ana No. 1000, 04440 Mexico City, Mexico

**Keywords:** Solar cells, Actuators

## Abstract

Improving the energy efficiency of robots remains a crucial challenge in soft robotics, with energy harvesting emerging as a promising approach to address it. This study presents a functional soft robotic composite called OPV-DEA, which integrates flexible organic photovoltaic (OPV) and dielectric elastomer actuator (DEA). The composite can simultaneously generate electrostatic bending actuation and harvest energy from external lights. Owing to its simplicity and inherent flexibility, the OPV-DEA is poised to function as a fundamental building block for soft robots. This study aimed to validate this concept by initially establishing the fabrication process of OPV-DEA. Subsequently, experimental samples are fabricated and characterized. The results show that the samples exhibit a voltage-controllable bending actuation of up to 15.6° and harvested power output of 1.35 mW under an incident power irradiance of 11.7 mW/cm^2^. These performances remain consistent even after 1000 actuation cycles. Finally, to demonstrate the feasibility of soft robotic applications, an untethered swimming robot equipped with two OPV-DEAs is fabricated and tested. The robot demonstrates swimming at a speed of 21.7 mm/s. The power consumption of the robot is dominated by a high-voltage DC-DC converter, with a value approximately 1.5 W. As a result, the on-board OPVs cannot supply the necessary energy during locomotion simultaneously. Instead, they contribute to the overall system by charging a battery used for the controller on board. Nevertheless, these findings suggest that the OPV-DEA could pave the way for the development of an unprecedented range of functional soft robots.

## Introduction

In recent years, the advancement in smart material technologies has gained significant attention, primarily owing to their wide applications in the field of soft robotics, ranging from compliant manipulators^1^ and strain sensors^2^ to mobile and autonomous robots^3,4^. However, enhancing the energy efficiency of soft robotic systems, including those utilizing smart materials, poses a significant challenge. One possible approach to address this issue is the implementation of energy harvesting functionalities^5^.

In this context, flexible organic photovoltaics (OPVs) can emerge as a key component for energy harvesting in soft robots. Their potential lies in their advantageous features, such as high flexibility because of their thin nature (typically ~ 3 µm thick), lightweight, and scalable architecture^6^. Additionally, OPVs can harvest energy from solar or artificial sources through the photovoltaic effect. They exhibit a high power-per-area, high power-per-weight, and excellent deformability, indicating that they can be compressed and stretched, while retaining even up to 87.3% retention of the initial performance after 5000 compressing–stretching cycles, with up to 33% of compression rate^7^. However, despite these features, almost no progress has been made in integrating OPVs into smart material-based soft robots. This may be attributed to the difficulty in the placement and design of OPVs with fitting into the shape and structure of smart materials or the robots.

Here, we propose the integration of OPVs into a smart material that acts as a functional composite capable of harvesting energy and generating actuation within one single element. This composite is expected to act as a building block for soft robots. As a smart material for such composite, we focused on the use of dielectric elastomer actuators (DEAs)^8–10^, a class of electroactive polymers, that consists mainly of a thin elastomer membrane sandwiched between two complaint electrodes, thus forming a “soft capacitor”. Under the application of a voltage (typically in the kV range), an electrostatic pressure is generated between the electrodes, resulting in an electrostatic actuation where the thickness of the elastomer membrane reduces, and its area expands. This phenomenon can be utilized to generate mechanical movement in a plane direction. It is known that DEAs possess desirable features for soft robotics, such as large deformation (e.g., strain more than 100%^11^), fast response time (e.g., less than 1 ms^11^), lightweight, and high energy density (e.g., 1.36 MJ/m^311^), coupled with their fast and cost-effective fabrication^12^. In addition, DEAs enable the self-sensing of their deformations^13,14^. The sheet-like structure of DEAs, resembling that of OPVs, facilitates easy integration and design flexibility. Moreover, DEAs exhibit diverse actuator configurations, enabling the creation of functional composites tailored for various types of soft robots. To the best of our knowledge, the integration of OPV-DEA has never been attempted before.

**Figure 1:**
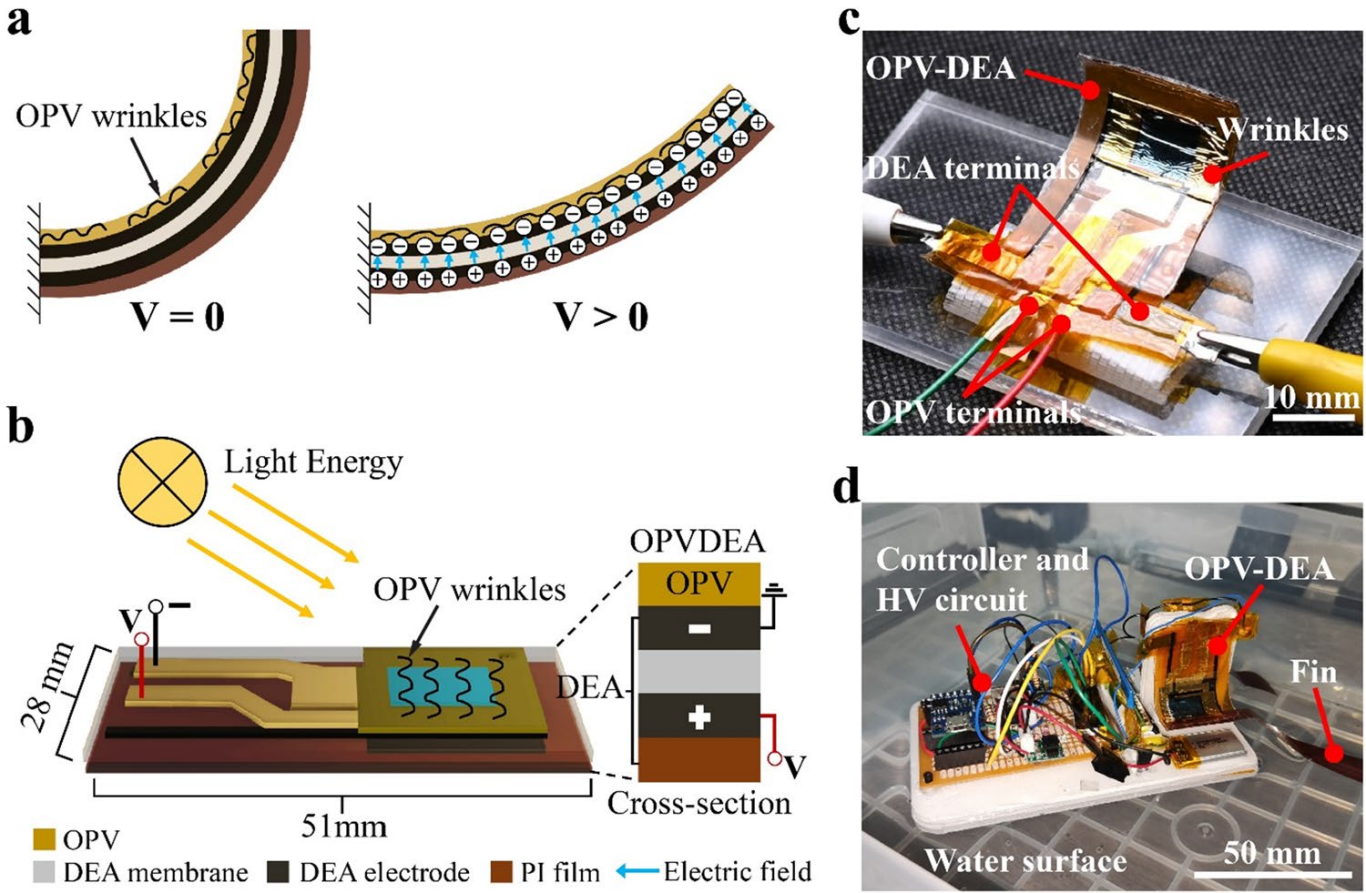
The organic photovoltaic (OPV) and dielectric elastomer actuator (DEA), namely OPV-DEA, developed in this study. (**a**) Actuation principle of OPV-DEA, exhibiting a bending motion toward a flat shape when voltage is applied. (**b**) Cross-section of the OPV-DEA, where an OPV is attached to a pre-stretched DEA. (**c**) Fabricated OPV-DEA with a default (zero voltage) bending state. (**d**) Swimming robot floating over water, with two OPV-DEAs that can provide mechanical actuation for moving the fins and subsequent locomotion, as well as energy harvesting.

The anticipated advantages of the functional soft robotic composite proposed in this study, namely OPV-DEA (Fig. 1), stem from the shared structure of OPV and DEA, which is characterized by planarity, thinness, and flexibility. This study aimed to validate the concept of OPV-DEA by establishing the fabrication process, characterizing experimental samples regarding actuation and energy harvesting performances, and integrating them into a soft robot. The results show the successful implementation of the concept, revealing the simultaneous actuation and energy-harvesting capabilities of OPV-DEA. Additionally, the locomotion of a swimming robot equipped with OPV-DEA is achieved.

## Results and discussion

The main functionalities of OPV-DEA are simultaneous voltage-controllable actuation and energy harvesting. To realize this concept, we laminated an OPV, a DEA, and other substrates sequentially using a layer-by-layer process. Moreover, we employed dielectric elastomer minimum energy structures (DEMESs), which is a configuration of DEA^15,16^. This configuration enables the generation of out-of-plane deformations by attaching a pre-stretched DEA onto a flexible substrate and it has been applied to various robots, such as a gripper^17^, crawling robot^18^, and fish robot^19^. Figures 1a and b illustrate the structure of OPV-DEA and Fig. 1c shows the fabricated OPV-DEA. As shown in the image, an OPV is placed on the top of a pre-stretched DEA, which is attached to a flexible substrate. Because the OPV is attached to the DEA in a flat state, wrinkles were formed in the OPV in the initial bending state (i.e., zero actuation voltage). These wrinkles enabled its bending deformation despite its inextensibility. Owing to the DEMES configuration, when a voltage was applied to the DEA, the entire structure exhibited a bending motion toward the flat shape. This bending motion enabled the integration of OPV-DEA with a soft robot capable of moving on the surface of water, where OPV enhanced its run time by harvesting energy from the surrounding environment (Fig. 1d).

**Figure 2:**
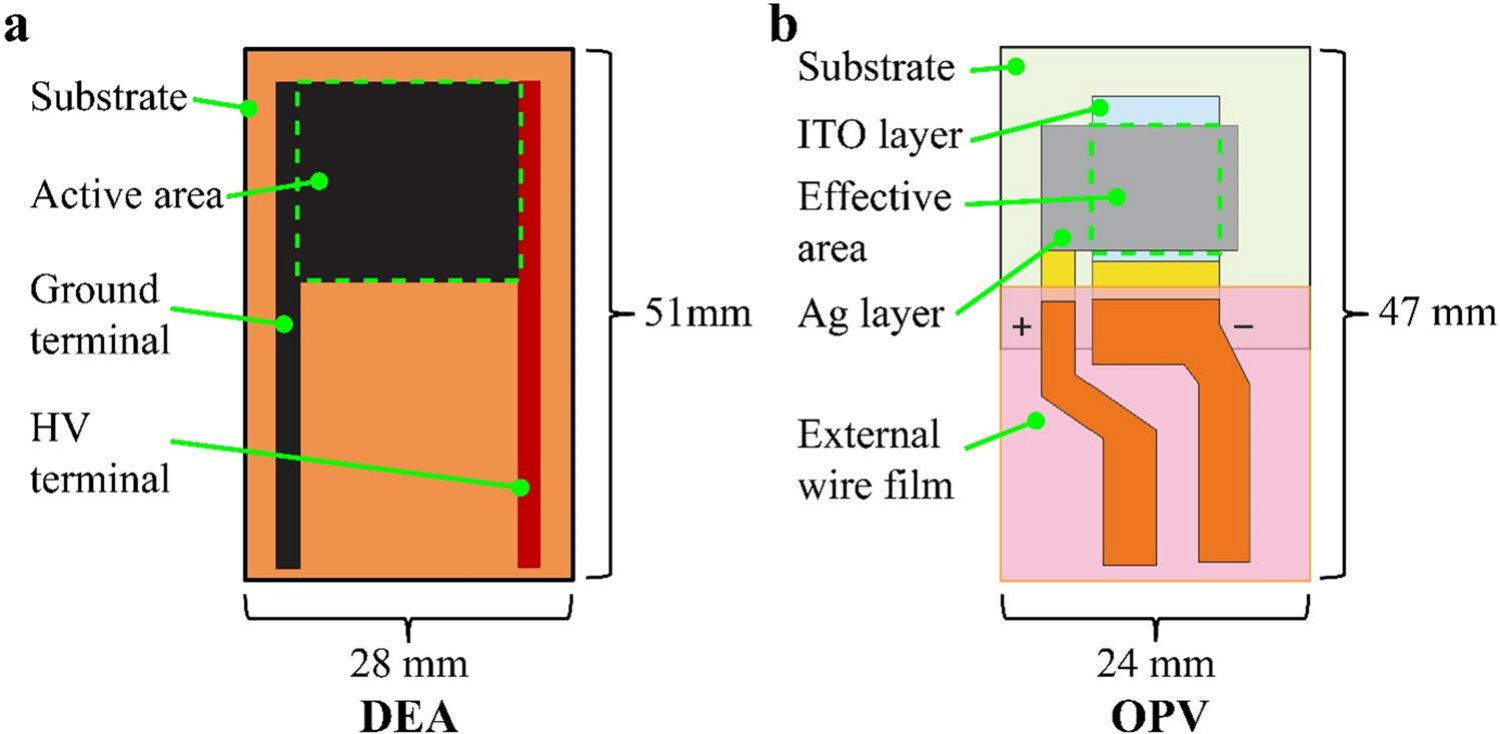
Geometry of OPV-DEA. (**a**) DEA geometry where the active area is located at the top center. (**b**) OPV geometry showing its effective area located at the top center (overlapping the DEA ground electrode).

To integrate OPV and DEA, there are several design factors, such as geometry, actuator configuration, and terminal accessibility, which should be considered. This integration should be capable of providing a mechanical output without obstructing the energy harvesting capability and vice versa. Thus, in this study, we selected a simple and straight design for the OPV-DEA with a main rectangular frame of 28 mm × 51 mm (width × length; Fig. 2). For the DEA part, a squared active area (area in which electrodes are overlapped) of 20 mm × 20 mm was employed, and this was located at the top center of the device, while the two terminals were designed to pass through both edges to avoid any electrical short circuit due to high voltages (Fig. 2a). The determined outline dimension of OPV was 24 mm × 24 mm (Fig. 2b). The effective area of the OPV consisting of the indium tin oxide (ITO) and silver (Ag) layers, which was located at the center of the DEA’s active area, was 10 mm × 10 mm. The terminals of OPV are designed such that they were located at the center to avoid any short circuit and electrical breakdown against the DEA part.

**Figure 3:**
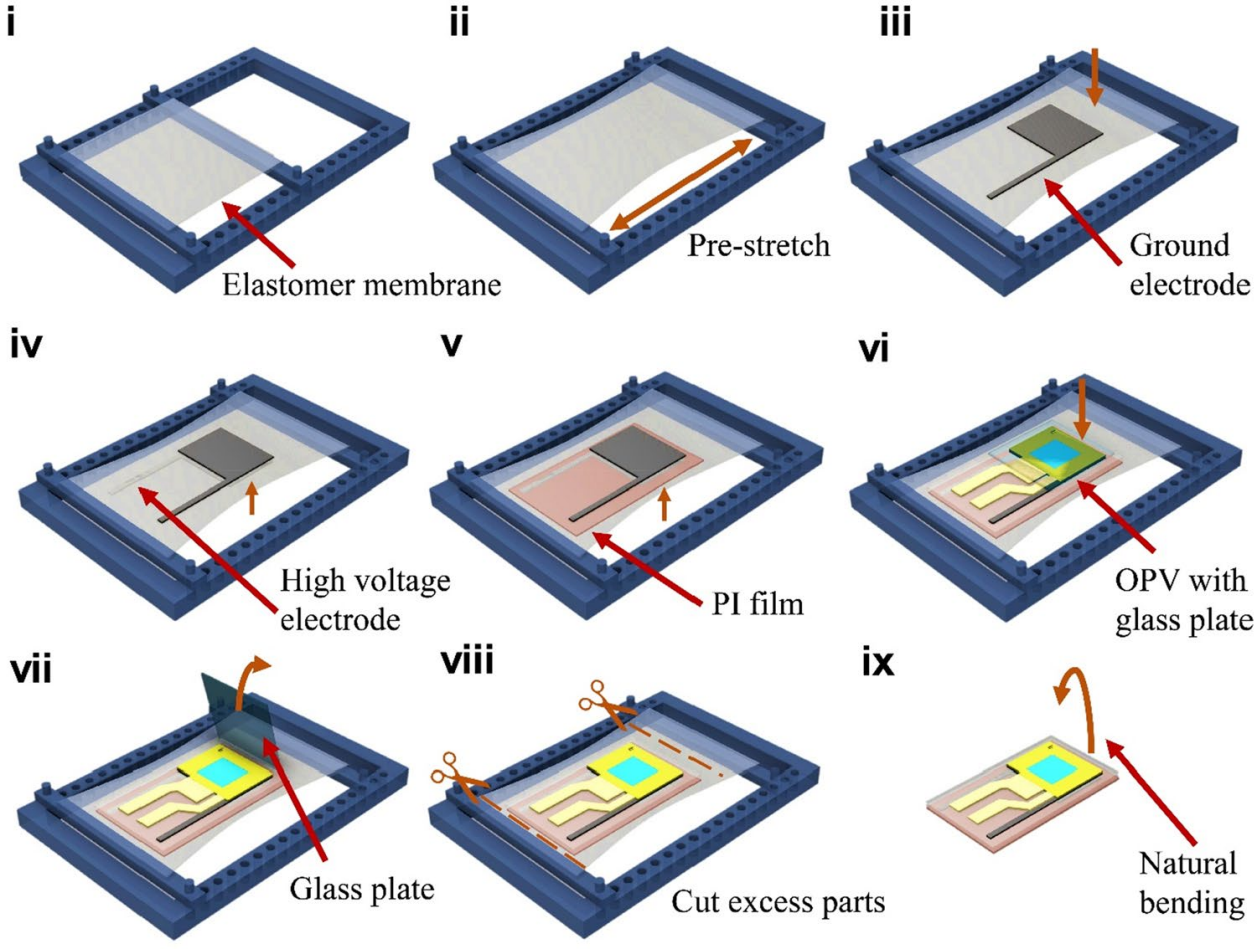
Fabrication process of OPV-DEA. (**i**) Elastomer membrane is fixed to the acrylic bracket. (**ii**) With the aid of the acrylic bracket, the membrane was prestretched. (**iii**) Ground electrode was attached to the membrane. (**iv**) High voltage electrode was attached to the other side of the membrane. (**v**) Pre-cut polyimide (PI) film substrate is attached to the bottom of the assembly. (**vi**) OPV with the glass plate is attached to the top of the assembly. (**vii**) Glass plate is removed from the assembly. (**viii**) The excess parts is cut using a pair of scissors. (**ix**) the final OPV-DEA is obtained.

The fabrication of OPV-DEA, which is summarized in Fig. 3, mainly involves the lamination of the DEA and OPV, beginning from the formation of the DEA part. An 0.5 nm-thick acrylic dielectric elastomer membrane (VHB 4905, 3 M) with initial outline dimensions of 31.8 mm (length) × 31.8 mm (width) was pre-stretched to a ratio of 1.6 and held between two acrylic supports (Fig. 3i-ii). Compliant electrodes made of a conductive acrylic elastomer (thickness = 33 µm; ARcare 90,366, Adhesives Research) were attached to both sides of the elastomer, after which a 125 µm-thick polyimide (PI) film was placed on the elastomer as the flexible substrate ((Fig. 3iii-v), forming the DEA part. The adhesive nature of the acrylic elastomers enabled the lamination of the layers without the need of additional glue and enabled rapid fabrication. In the previously-described steps, both the dielectric and conductive elastomers were cut using a laser machine (Speedy 300, Trotec), whereas the PI film was cut manually using a cutter knife. Thereafter, an OPV prepared on a glass plate was placed on top of the DEA. Subsequently, the glass plate was removed carefully using a plastic tool (Fig. 3vi-vii). Lastly, using a pair of scissors, the entire assembly was released, and all the unnecessary parts were cut, resulting in a final OPV-DEA with an initial bending angle (Fig. 3viii-ix). Further details on the fabrication process of the OPV part can be found in the Method Section.

The OPV-DEA offers multifunctionality, serving as an energy harvester and actuator. The OPV part harvests energy from external lights, providing a renewable power source. Conversely, the DEA part allows for voltage-controllable actuation. To elucidate the characteristics of these functionalities, we characterized the OPV-DEA across several performance metrics.

**Figure 4:**
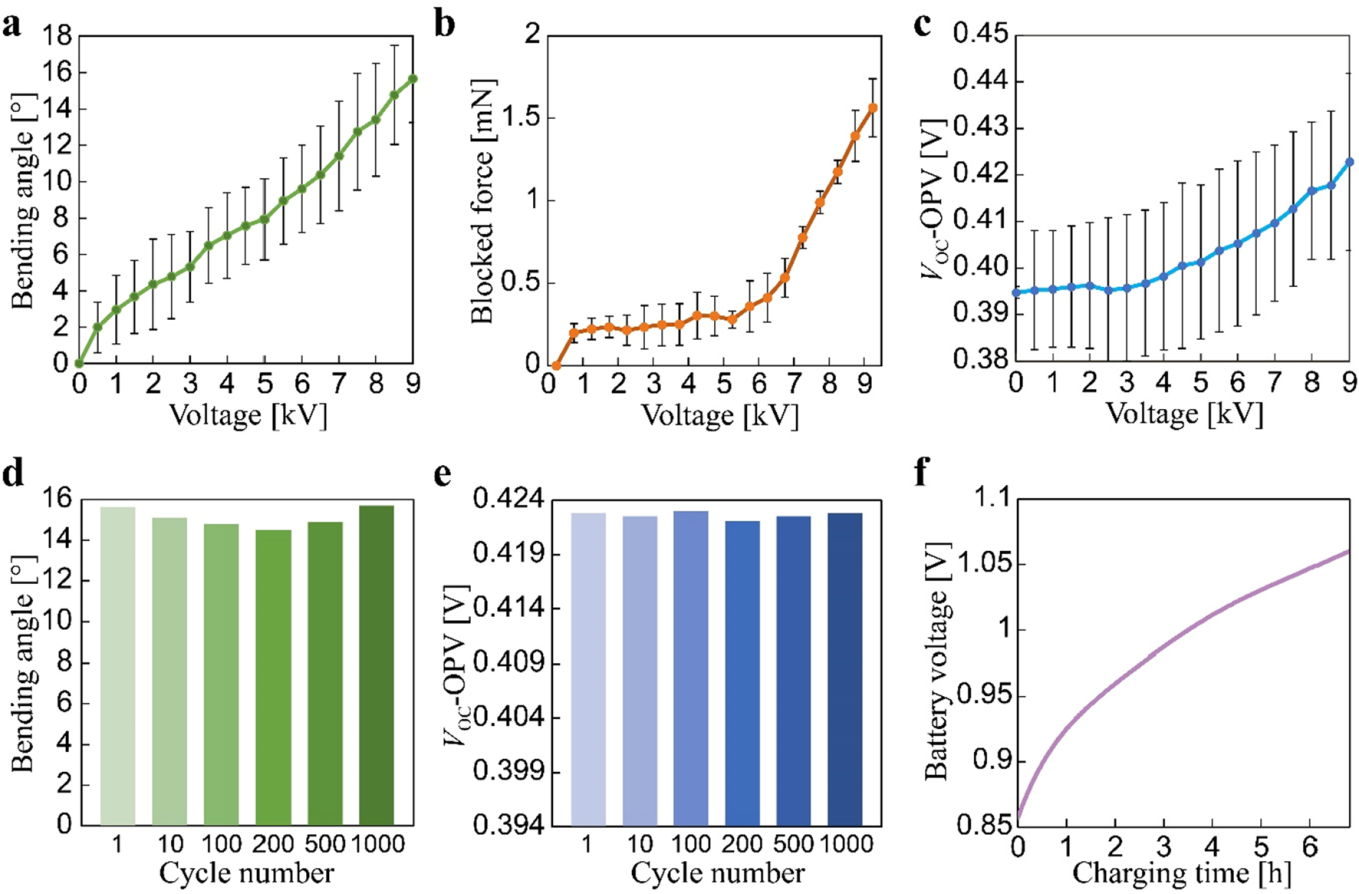
Characterization results of the OPV-DEA. (**a**) Bending angle as a function of the applied voltage. (**b**) Blocked force as a function of the applied voltage. (**c**) The open circuit voltage (*V*_OC_) of the OPV as a function of the applied voltage. Data in (**a**)–(**c**) are the average of four samples. (d) Bending angle under cyclic actuation (0 and 9 kV). (**e**) The *V*_OC_ values of the OPV under cyclic actuation (0 and 9 kV). (**f**) Battery voltage as a function of the charging time.

The evaluation of the actuation functionality involves assessing the actuated bending angle and blocked force regarding the applied voltage. Figure 4a depicts the measured data, indicating that the actuated angle increases with voltage, reaching a peak value of 15.6° at 9 kV (Supplementary Video S1). The actuated force plotted in Fig. 4b exhibited a correlation with voltage, rising to a value of 1.6 mN at 9 kV. This value is within the same order of magnitude as forces observed for other DEA-based actuators of similar type (i.e., DEMESs) reported in the literature^15–17^.

To evaluate the energy harvesting functionality, we initially assessed the electrical characteristics of a non-actuated OPV-DEA, including output voltage and current, fill factor (FF), and power conversion efficiency (PCE). The device demonstrated an open-circuit voltage (*V*_OC_) of 0.395 V, a short-circuit current (*I*_SC_) of 5.8 mA, and an FF of 55.5%, resulting in a power output of 1.35 mW and PCE of 11.4%, under an incident power irradiance of 11.7 mW/cm^2^. These results are consistent with those from other similarly structured stand-alone OPVs reported in the literature^7^. The interaction between the OPV part and DEA part is pivotal for OPV-DEA functionality. Since the active and effective areas of the DEA and OPV parts are located in the region of the device, respectively, the effective area of the OPV part is anticipated to unfold when the device is actuated towards the flat state (Fig. 1a). This may result in an increase in harvested energy as the overall effective area expands with the flattening of the device. To investigate this behavior, we proceeded to characterize the open-circuit voltage *V*_OC_ of the OPV-DEA under voltage-controlled deformation and an incident power irradiance of 11.7 mW/cm^2^. As shown in Fig. 4c, as the applied voltage for the DEA part increases, the device gradually flattens, resulting in a subsequent rise in the measured *V*_OC_ value. The device exhibited an initial value of 0.395 V, reaching a peak of 0.423 V, reflecting a total increase of 28 mV. This finding indicates that the relationship between the OPV part and DEA part can be beneficial and does not impede OPV-DEA operation.

Given that the OPV-DEA is comprised of several film materials laminated together, it is crucial to assess its reliability. Therefore, we examined its actuation and energy harvesting performance through repeated cycles of up to 1,000. The energy harvesting performance was measured via *V*_OC_, while the actuation performance was determined through the bending angle. The result consistently showed stable outputs throughout the tested actuation cycles (Fig. 4d,e). Significantly, the changes in actuation and energy harvesting performance during the cycles were marginal, registering only 5.1 and 0.21%, respectively. This underscores the high robustness of OPV-DEA, making it well suited for soft robotic applications that often involve cyclic action.

**Figure 5:**
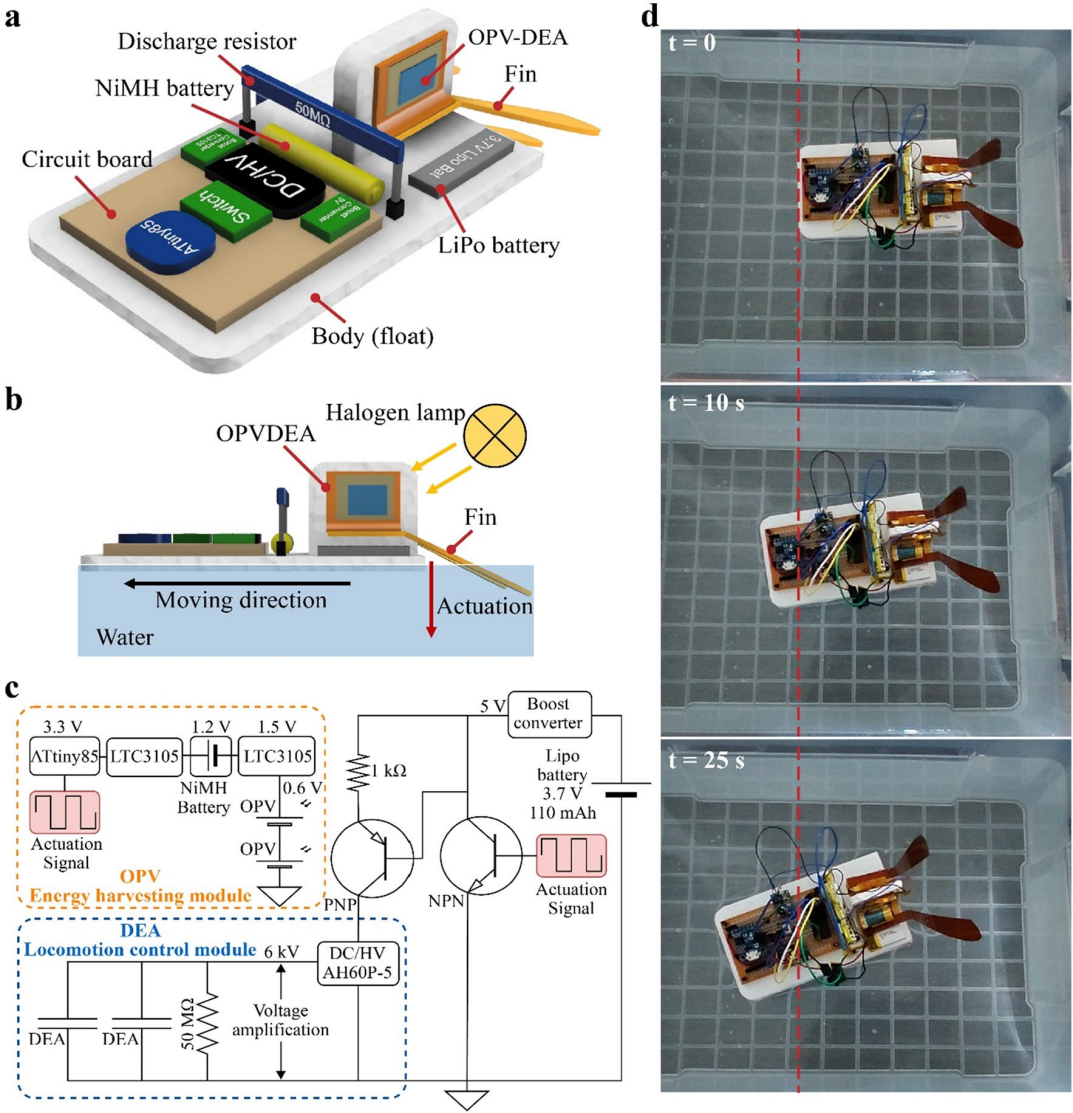
Swimming robot based on the fabricated OPV-DEA. (**a**) Structure of the robot. It employs two OPV-DEAs, which are connected to an elastic fin made of PI film. (**b**) Working principle of the robot: the energy of the external light (halogen lamp) gets absorbed by the OPV-DEAs and is then utilized to drive the actuators, pushing the fin downwards and propelling the robot forward. (**c**) Electrical circuit diagram of the robot system, which is divided into two modules responsible for locomotion and energy harvesting. (**d**) Sequence of the swimming of the robot.

To demonstrate the applicability of the OPV-DEAs in soft robotics, we integrated them into an untethered swimming robot (Fig. 5a). The robot is self-contained, comprising two OPV-DEAs connected to elastic fins made of a laser-cut PI film, a high-voltage DC/DC converter (AH60P-5, XP power), circuit board, microcontroller (AtTiny85), main battery (LiPo 3.7 V, 110 mA), and an auxiliary battery (NiMH 1.2 V). The robotic system consists of two main modules: a locomotion module and an energy-harvesting module. The former facilitates the bending actuation of the OPV-DEAs, which in turn drives the oscillation of the fins, generating thrust forces that propel the robot forward (Fig. 5b). The latter module harvests energy from external lights to charge the auxiliary battery, which powers the microcontroller.

Figure 5c shows that the main power source for the energy-harvesting module consists of two OPVs arranged in series within the OPV-DEAs. This array registered a *V*oc of 0.66 V, a short-circuit current (${I}_{SC}$) of 5.7 mA, and FF of 40.0%. Consequently, it provided a power output of 1.5 mW and a PCE of 13.5% when exposed to an incident power irradiance of 11.7 mW/cm^2^. To charge the auxiliary battery (NiMH 1.2 V) effectively, the power output was boosted by incorporating a boost converter circuit (LTC3105). This circuit is specifically designed to work with small solar cells, effectively increasing the voltage to 1.5 V.

We performed a charging test with the robot in a non-actuated state, observing an initial charging current of 91 µA. The voltage steadily increased, raising the battery level from 0.85 V to a final level of 1.05 V under an incident power irradiance of 11.7 mW/cm^2^ (Fig. 4f and Supplementary Video S1). The microcontroller (ATtiny85) in the energy-harvesting module controlled the operations of the robot. However, this microcontroller requires a minimum operation voltage of 3.3 V. To elevate the output voltage of the OPV-charged auxiliary battery to the necessary level, we connected another booster circuit (LTC3105) between the microcontroller and auxiliary battery (Fig. 5c). Upon activation, the microcontroller produces a square signal to actuate the OPV-DEAs. The locomotion module is powered by the main battery (LiPo battery 3.7 V, 110 mA), which was previously charged using a DC power supply (RD6018, Riden). Figure 5c shows that the main battery was connected to a miniaturized high-voltage DC-DC converter (AH60P-5, XP power) that elevates the output up to 6 kV.

The experiment conducted in a water tank revealed that the swimming speed of the robot was 21.7 mm/s when operated at 6 kV with a driving frequency of 2.5 Hz (Fig. 5d and Supplementary Video S1). This experiment was performed using the energy harvested and stored in the auxiliary battery from the previous test. The high-voltage DC-DC converter dominates the overall power consumption of the robot, which is approximately 1.5 W. This value is significantly higher than the output of the on-board OPVs (1.5 mW). Consequently, the DEAs in the robot cannot be directly powered by the OPVs. Instead, they were used to charge the auxiliary battery that powers the controller. This setup underscores the utility of OPVs. Without a signal from the controller, which is powered and operated by the OPVs, the robot would be incapable of executing any controlled movements. Furthermore, the OPVs supplying energy to the controller improve the efficiency of the robot. A longer operation time for the controller ensures the standby time of the robot, which is often crucial for tasks such as remote communication and fixed-point observation during missions. Therefore, the observed swimming locomotion of the robot illustrates the versatility of OPV-DEA, serving as both an energy harvester for the power and a voltage-controllable actuator for robot locomotion. This demonstrates its practical applicability in soft robotic systems.

## Concluding remarks

In this study, we proposed the OPV-DEA as a multifunctional composite to address a crucial challenge in soft robotics: improving energy efficiency through energy harvesting. The OPV-DEA is capable of simultaneously harvesting energy from external lights and generating voltage-controllable actuation. To prove the concept of OPV-DEA, we first established the fabrication process based on the lamination of OPV, DEA, and substrate. We then fabricated and characterized experimental samples, observing bending actuation and blocked force of up to 15.6° and 1.6 mN, respectively. We also observed a *V*_OC_ of 0.395 V, an *I*_SC_ of 5.8 mA, and FF of 55.5%, resulting in a power output of 1.35 mW and PCE of 11.4% under an incident power irradiance of 11.7 mW/cm^2^. These performances were maintained even after 1,000 actuation cycles. Lastly, to demonstrate the feasibility of soft robotic applications, we integrated two OPV-DEAs to form an untethered swimming robot capable of moving across the water surface at a speed of 21.7 mm/s.

Given that DEAs offer diverse actuator configurations, future work should investigate OPV-DEAs under various architectures. Furthermore, in the future, not only DEAs but also other smart materials with thin nature, such as ionic polymer–metal composites^20^ and liquid crystal elastomer actuators^21^, could be integrated into OPVs. In addition, high stretchability of OPVs may enable integration with fluidic elastomer actuators^22^ that rely on the inflation of the actuator structure for their actuation. Applying these novel OPV-DEAs into soft matter systems may open up the path into the fabrication of an unprecedented range of functional soft robots.

## Methods

### Materials for the fabrication of the flexible organic photovoltaics

The precursor (ECRIOS VICT-Cz) used for the fabrication of the transparent polyimide substrate was obtained from Mitsui Chemicals. The fluorinated polymers (Novec1700, 3 M) and their solvents (Novec 7100, 3 M) were purchased from 3 M. Zinc acetate dehydrates, ethanolamine, 2-methoxyethanol, and chlorobenzene were purchased from FUJIFILM Wako Pure Chemical Corporation. Ethoxylated polyethyleneimine (PEIE) and 1-chloronaphthalene were purchased from Sigma Aldrich. Poly[4,8-bis(5-(2-ethylhexyl) thiophen-2-yl) benzo[1,2-b;4,5-b’] dithiophene-2,6-diyl alt-(4-octyl-3-fluorothieno[3,4-b] thiophene)-2-carboxy-late-2–6-diyl] (PBDTTT-OFT) was obtained from TORAY. 2,2’-((2Z,2’Z)-(((4,4,9,9-tetrakis(4-hexylphenyl)-4,9-dihydro sindaceno[1,2-b:5,6-b’]dithiophene-2,7-diyl)bis(4-((2-ethylhexyl)oxy)thiophene-5,2 diyl))bis(methanylylidene))bis(5,6-difluoro-3-oxo-2,3-dihydro-1H-indene2,1diylidene)) dimalononitrile (IEICO- 4F) was purchased from 1-Material. All the materials were used as received without further purification.

### Fabrication of the flexible organic photovoltaics

This process is based on a previously reported process^23^. First, the glass substrates were treated with oxygen plasma for 10 min at 300 W (PC-300, Samco). Thereafter, the fluorinated polymer layer (Novec 1700:7100 = 1:8) was spin coated (MS-B100, Miksa) on a 50 mm × 50 mm glass at 4000 rpm for 1 min. Next, the fluorinated glass substrate was heated in an inert oven at 80 °C for 10 min. Before spin coating the transparent polyimide precursor, the glass/fluorinated polymer layer was treated with oxygen plasma for 5 s at 50 W. The precursor was spin coated onto the substrate at 2000 rpm for 1 min to form a film with a thickness of approximately 2.4 µm. The transparent polyimide film was cured through an imidisation reaction at 250 °C for 2 h in an inert oven under nitrogen atmosphere (DN411I, Yamato Scientific). Thereafter, an indium tin oxide (ITO) transparent electrode with a thickness of 100 nm was deposited on the substrate using a sputtering machine (SIH-1010, ULVAC, Inc.). The ITO electrode was patterned using photolithography, and 3.5 nm-thick Cr and 100 nm-thick Au layers were deposited on the ITO electrode as supporting wires. Subsequently, PEIE chelated with Zn^2+^ (PEI–Zn) was used as the electron transport layer 28,37. The PEI–Zn precursor solution was prepared by dissolving 70 mg of zinc acetate dihydrate in 1 wt% PEIE 2-methoxyethanol. To form the PEI–Zn film, the precursor solution was spin coated at 3500 rpm for 45 s and subsequently thermally annealed at 180 °C for 30 min in air. Thereafter, PBDTTT-OFT (10 mg) and IEICO-4F (15 mg) in chlorobenzene (970 µL) were heated at 70 °C for 2 h, after which the additive, 1-chloronaphthalene (30 µL), was added into the solution and stirred at 70 °C for 5 min. The solution was spin coated in ambient air at 1400 rpm for 60 s to form a bulk heterojunction photoactive layer. Unnecessary areas were carefully removed using a cotton swab soaked in chloroform. The dried samples were placed in a vacuum evaporator, and a hole-transporting layer of molybdenum oxide (MoOX, 7.5 nm) and an Ag anode (100 nm) were sequentially deposited through thermal evaporation at < 3 × 10^−4^ Pa. Lastly, a 1 µm-thick parylene layer was deposited through chemical vapor deposition to form a passivation layer. The fabricated solar module exhibited a one-cell design with an effective area of 10 × 10 mm.

### Characterization of the OPV-DEA

As shown in the experimental setup in Figure S1, the characterization was conducted by applying a voltage of up to 9 kV to the actuator with an 0.5 kV increase rate. The voltage was supplied from a high-voltage DC-DC converter (CB101, XP Power). A light source was positioned above the actuator (WP-960, Photo Lamp). The actuation angle as a function of the applied voltage was obtained by measuring the displacement of the actuator tip, which was performed using a camera (D3500, Nikon), followed by image processing. To characterize the bending–V_*OC*_ relationship, the energy harvesting values at each interval were measured using a digital multimeter (2100, Keithley). We measured a non-actuated OPV-DEA using two 100 W Halogen bulbs (OSRAM 64627) powered by a DC power supply (RD6018, Riden), which generated 11.7 mW/cm^2^ of power irradiance.

The blocked force of the actuator was measured using a load cell sensor (FS1M-0.1NB, THK Precision). The experimental set up is shown in Figure S2. To identify the accurate normal force, the load cell was placed such that it is in contact with the actuator’s tip. Up to 9 kV of voltage was applied to the actuator with an 0.5 kV increase rate. The cyclic test of the OPV-DEAs was conducted based on the setup shown in Figure S1: a sample was actuated repeatedly through the high voltage DC-DC converter and a function generator (Matsusada, eK-FGJ). The bending angle and the *V*_OC_ of the OPV-DEAs as a function of the applied voltage were obtained at actuation cycles of 1, 10, 100, 200, 500 and 1000.

### Charging test of the battery on board the robot

A 1.2-V NiMH battery was discharged to 0.85 V using an adjustable constant current electronic load (150W DIY300W). The photovoltaic of each OPV-DEA was connected in series, and then to the input side of a boost converter circuit (LTC3105), while the output side was connected to the terminals of the 1.2-V NiMH battery. A microcontroller (Arduino Uno Rev 3, Arduino) was connected in parallel to the battery, and then to a PC. This enabled voltage sensing and data processing. Two 100 W Halogen bulbs (OSRAM 64,627) were set up around the robot and powered by a DC power supply (RD6018, Riden), resulting in a power irradiance of 11.7 mW/cm^2^. This induced a *V*_OC_ of 0.6 V on the OPV-DEA. The LTC3105 enhanced this output to 1.5 V. Thereafter, electric current was flowed to the battery to initiate the charging process. The experiment was conducted until the battery reached a voltage of 1.05 V.

### Supplementary Information

Supplementary Figures.

Supplementary Video 1.

Supplementary Legends.

## Data Availability

All data that support the plot within this paper and other findings of this study are available from the corresponding author upon reasonable request.
