# Supplementary material for: Functional soft robotic composites based on organic photovoltaic and dielectric elastomer actuator
Source: Sci Rep. 2024 Apr 30;14:9953. doi: 10.1038/s41598-024-60899-6 (PMC11061127; doi:10.1038/s41598-024-60899-6)
Supplement: Supplementary file 1 — Supplementary Figures. [file 41598_2024_60899_MOESM1_ESM.docx]

Supplementary Information

**Functional soft robotic composites based on organic photovoltaic and dielectric elastomer actuator**

Ahmed Miguel Román Abolhosen, Kenjiro Fukuda, Hernández González Leobardo and Jun Shintake


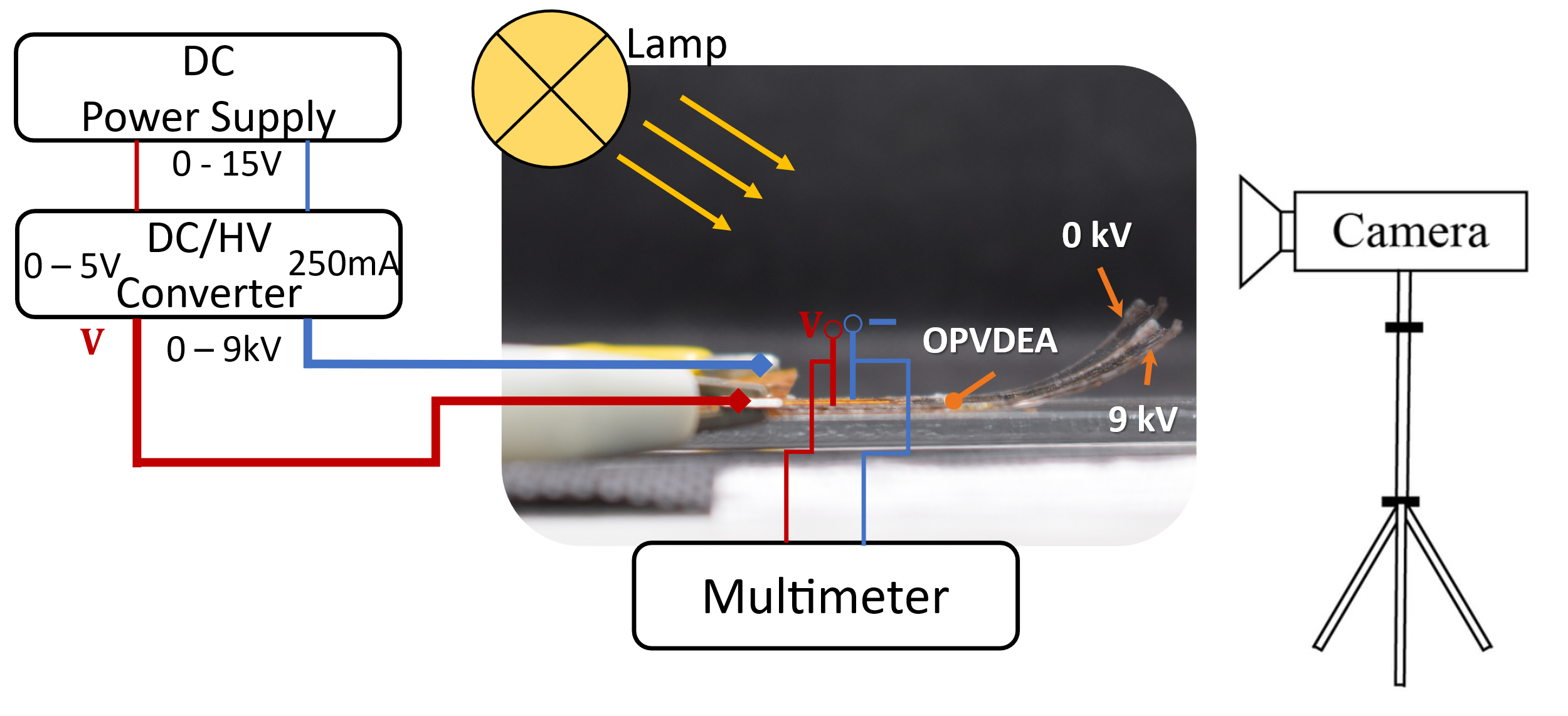


**Figure S1.** Experimental setup used for the characterization of OPV-DEA in terms of bending angle (actuation) and energy harvesting.


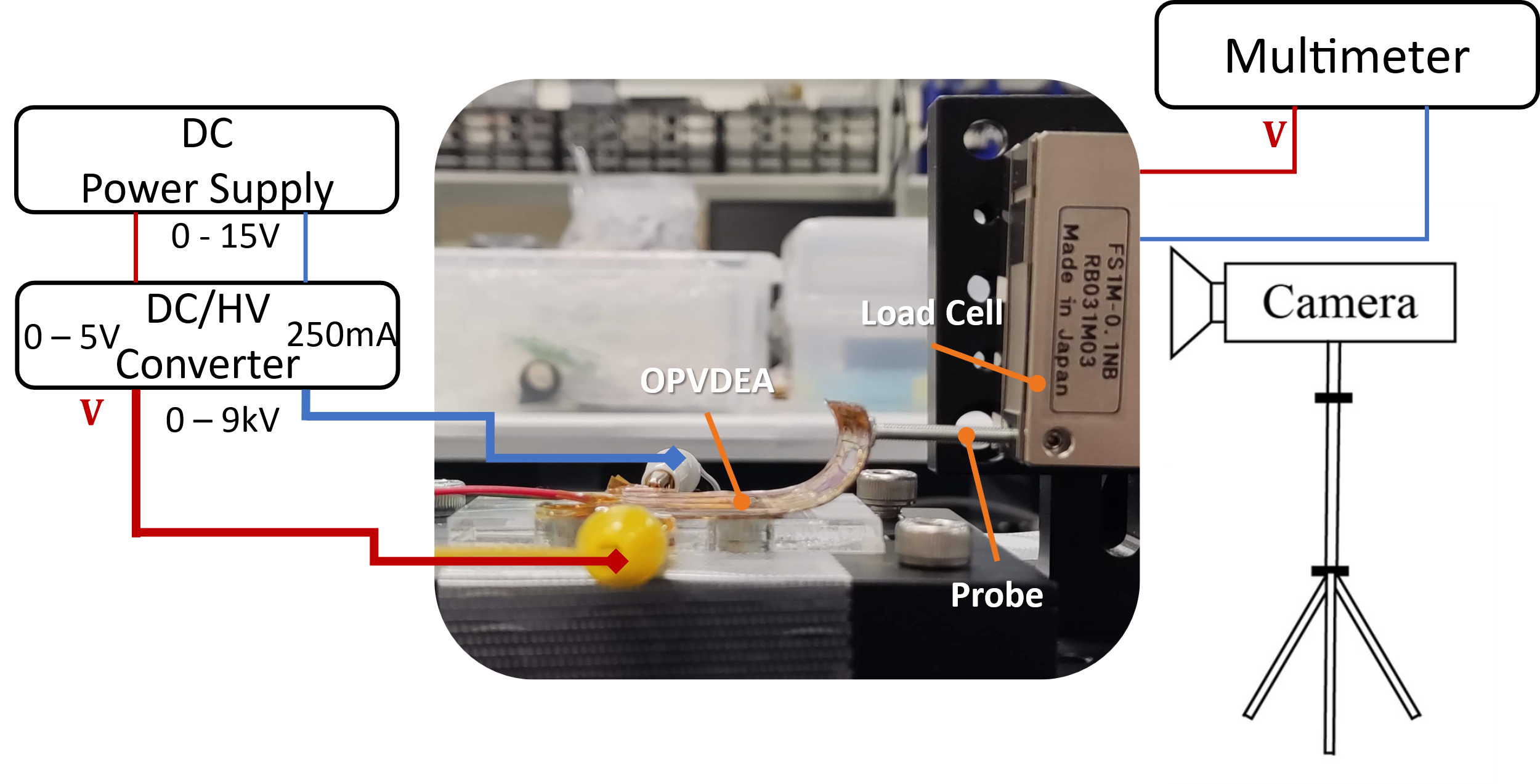


**Figure S2.** Experimental setup used for the characterization of OPV-DEA in terms of blocked force.
