# Supplementary material for: Functional soft robotic composites based on organic photovoltaic and dielectric elastomer actuator
Source: Sci Rep. 2024 Apr 30;14:9953. doi: 10.1038/s41598-024-60899-6 (PMC11061127; doi:10.1038/s41598-024-60899-6)
Supplement: Supplementary file 3 — Supplementary Legends. [file 41598_2024_60899_MOESM3_ESM.docx]

Operation of the OPV-DEA and locomotion of the robot.
